# Strategies to Maintain Redox Homeostasis in Yeast Cells with Impaired Fermentation-Dependent NADPH Generation

**DOI:** 10.3390/ijms25179296

**Published:** 2024-08-27

**Authors:** Magdalena Kwolek-Mirek, Roman Maslanka, Sabina Bednarska, Michał Przywara, Kornelia Kwolek, Renata Zadrag-Tecza

**Affiliations:** 1 Institute of Biology, College of Natural Sciences, University of Rzeszow, 35-959 Rzeszow, Poland; rmaslanka@ur.edu.pl (R.M.); sbednarska@ur.edu.pl (S.B.); michal.przywara.24@gmail.com (M.P.); 2 Department of Plant Biology and Biotechnology, Faculty of Biotechnology and Horticulture, University of Agriculture in Krakow, 31-425 Krakow, Poland; kornelia.kwolek@gmail.com

**Keywords:** NADPH generation, NADPH/NADP^+^ ratio, glucose-6-phosphate dehydrogenase, 6-phosphogluconate dehydrogenase, aldehyde dehydrogenase 6, GSH/GSSG ratio, redox homeostasis, pH homeostasis, acetic acid, yeast

## Abstract

Redox homeostasis is the balance between oxidation and reduction reactions. Its maintenance depends on glutathione, including its reduced and oxidized form, GSH/GSSG, which is the main intracellular redox buffer, but also on the nicotinamide adenine dinucleotide phosphate, including its reduced and oxidized form, NADPH/NADP^+^. Under conditions that enable yeast cells to undergo fermentative metabolism, the main source of NADPH is the pentose phosphate pathway. The lack of enzymes responsible for the production of NADPH has a significant impact on yeast cells. However, cells may compensate in different ways for impairments in NADPH synthesis, and the choice of compensation strategy has several consequences for cell functioning. The present study of this issue was based on isogenic mutants: Δ*zwf1*, Δ*gnd1*, Δ*ald6*, and the wild strain, as well as a comprehensive panel of molecular analyses such as the level of gene expression, protein content, and enzyme activity. The obtained results indicate that yeast cells compensate for the lack of enzymes responsible for the production of cytosolic NADPH by changing the content of selected proteins and/or their enzymatic activity. In turn, the cellular strategy used to compensate for them may affect cellular efficiency, and thus, the ability to grow or sensitivity to environmental acidification.

## 1. Introduction

Redox homeostasis is a balance between oxidation and reduction reactions, and to maintain it, cells have a well-coordinated system of antioxidant enzymes and low-molecular-weight compounds [1]. Glutathione (L-γ-glutamyl-L-cysteinylglycine; GSH) plays a crucial role in this system, mainly due to the thiol group of cysteine and its changes during redox reactions. GSH is synthesized in a two-step ATP-mediated reaction occurring in the cytosol. In the first step, catalyzed by γ-glutamylcysteine ligase (γ-GCS, product of *GSH1* gene), L-glutamate connects to L-cysteine. In the second step, the reaction of L-γ-glutamyl-L-cysteine and glycine is catalyzed by glutathione synthetase (GS, product of *GSH2* gene) [1,2,3,4]. In cells, glutathione exists in two redox states, as reduced glutathione (GSH) and as glutathione disulfide (GSSG), which is the oxidized form. The proportions of both forms depend on the growth conditions and type of cellular metabolism. During undisturbed growth of yeast cells, reactive oxygen species (ROS) are generated in the physiological range and play mainly a signaling role; therefore, GSSG is found at a very low level in comparison to the level of GSH. This is due to the activity of NADPH-dependent glutathione reductase (GR, product of *GLR1* gene) catalyzing the reduction of GSSG to GSH [5,6,7]. Given that GSH and GSSG constitute the major intracellular redox buffer, they are present in varying concentrations and ratios depending on the organism and the cellular compartment. The GSH/GSSG ratio in the total yeast cell extracts ranges from 30:1 to 100:1, which corresponds to the redox potential of the GSH/GSSG couple in the range of −220 to −232 mV [8,9]. The cytosolic GSH/GSSG redox potential is even more reducing than previously thought when measured with redox-sensitive fluorescent proteins: rxYFP (−289 mV) and roGFP2 (from −320 mV to −350 mV), which correspond to a GSH/GSSG ratio of approximately 3000:1 and 50,000:1, respectively [10,11,12]. However, this ratio can change significantly under stressful conditions, as shown by numerous studies [13,14,15]. GSH enables the maintenance of redox homeostasis through thiol–disulfide exchange reactions with cysteine-containing proteins but also plays a role as an electron donor for antioxidant enzymes. In addition, GSH plays a protective role against reactive electrophiles (such as ROS or xenobiotics) by reacting with them directly (non-enzymatically) or via glutathione S-transferases (GST) [1,2,4,7].

A crucial role of both glutathione in general and the intracellular GSH/GSSG redox potential has been emphasized for many years. Nevertheless, it should be taken into account that redox homeostasis may be influenced by other redox couples present in cells, including the reduced nicotinamide adenine dinucleotide phosphate (NADPH) and its oxidized form, NADP^+^. The NADPH/NADP^+^ ratio in eukaryotic cells is approximately 100:1, but in yeast cells, it ranges from 1:10 to 5:1, which corresponds to the redox potential of the NADPH/NADP^+^ couple from −290 to −340 mV [9,16,17]. However, the NADPH/NADP^+^ ratio changes under stress conditions [13,14,18]. NADPH is an essential electron donor and provides the reducing power for anabolic reactions and the redox balance. Moreover, it is particularly important for the supporting glutathione system under stress conditions. NADPH plays a role as a cofactor for glutathione reductase (GR) and thioredoxin reductase (TRR), which are required to maintain reduced glutathione and thioredoxin (TRX). Therefore, maintaining the proper pool of NADPH is crucial for the efficient reduction of glutathione and thus maintaining the optimal redox balance in cells [9,13]. NADPH is also effective in both scavenging free radicals and repairing biomolecule-derived radicals [19,20]. Furthermore, NADPH is used as a reductant in several reactions, including the biosynthesis of fatty acids, amino acids, and nucleotides but also in the detoxification of xenobiotics [21,22,23,24].

The main source of NADPH in the *Saccharomyces cerevisiae* yeast cells during growth on a medium with glucose is a pentose phosphate (PP) pathway in which glucose-6-phosphate is converted to ribulose-5-phosphate with the simultaneous reduction of two molecules of NADP^+^ to NADPH. These reactions are catalyzed by glucose-6-phosphate dehydrogenase (Zwf1p, G6PDH, product of *ZWF1* gene) and 6-phosphogluconate dehydrogenase (Gnd1p and Gnd2p, 6-PGD, product of *GND1* and *GND2* genes) [25,26,27]. An essential source of NADPH is also the NADP^+^-dependent oxidation of acetaldehyde to acetate via aldehyde dehydrogenase 6 (Ald6p, product of *ALD6* gene) [28].

Considering the crucial role of NADPH in maintaining intracellular redox homeostasis, it is important to provide new data concerning the possible crosstalk between the GSH/GSSG and NADPH/NADP^+^ redox couples, which is a burning issue in current redox biology research. Therefore, this study aimed to investigate (i) how the lack of enzymes responsible for NADPH production affects yeast cells; (ii) what strategies the cells use to compensate for these deficiencies; and (iii) whether it allows the cells to maintain the proper balance between the reduced and oxidized forms of the GSH/GSSG and NADPH/NADP^+^ redox couples. A comprehensive panel of molecular and biochemical analyses was used, including the gene expression level, protein content, and enzyme activity. The influence of the impaired generation of NADPH on redox homeostasis, growth of cells, pH homeostasis, and sensitivity to acetic acid was also examined. To assess the role of glucose and its metabolism in NADPH production in yeast cells, studies were performed after 12 h (fermentative metabolism) and 24 h of culture (condition after metabolic shift from fermentation toward respiration).

## 2. Results

### 2.1. Consequences of the Lack of Enzymes Responsible for Fermentation-Dependent NADPH Generation in Yeast Cells

**Figure 1:**
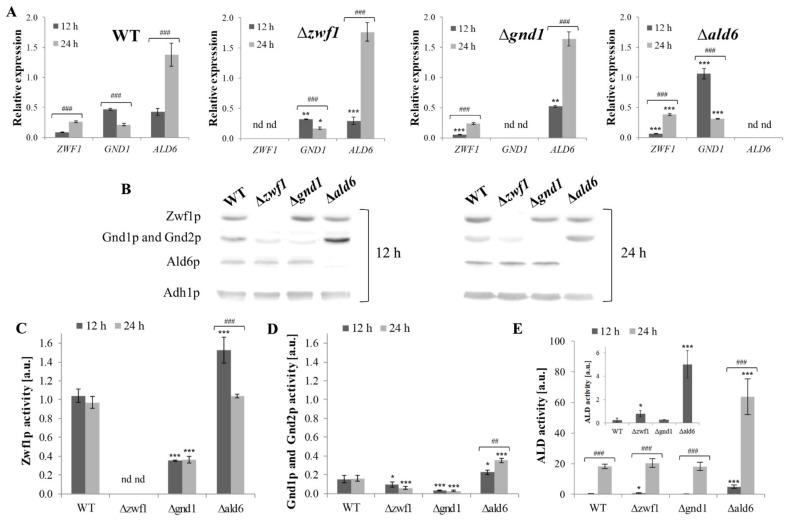
*ZWF1*, *GND1*, and *ALD6* gene expression, protein content, and enzyme activity in the wild-type (WT) and Δ*zwf1*, Δ*gnd1*, and Δ*ald6* mutant strains after 12 and 24 h of culture. *ZWF1*, *GND1*, and *ALD6* (**A**) gene expressions were determined by qPCR assay with TaqMan probes. The relative gene expression was calculated with the -ΔC_T_ method for comparison of the expression of different genes in the same strain. The Zwf1p, Gnd1p, and Ald6p proteins (**B**) were detected by immunoblotting assay with the primary antibodies: anti-glucose-6-phosphate dehydrogenase (1:2000), anti-6-phosphogluconate dehydrogenase (1:2000), and anti-yeast aldehyde dehydrogenase (1:4000), and with the horseradish peroxidase-conjugated secondary antibodies (1:10,000) with a chemiluminescent substrate. Adh1p was used as an internal control. The activity of Zwf1p (**C**), and the Gnd1p and Gnd2p (**D**) enzymes in the whole-cell protein extracts was determined spectrophotometrically by measuring the rate of NADP*^+^* reduction at 340 nm activity, considering the total activity of the PP pathway dehydrogenases as well as separately the 6-phosphogluconate dehydrogenase activity. The Zwf1p activity was calculated by subtracting the activity of Gnd1p and Gnd2p from the total PP pathway dehydrogenases activity. The aldehyde dehydrogenases activity (**E**) was determined with an Aldehyde Dehydrogenase Activity Colorimetric Assay Kit spectrophotometrically by measuring the absorbance using a Tecan Infinite 200 microplate reader at λ = 450 nm. The ALD activity was expressed per mg of protein. The small graph presents the ALD activity after 12 h for detailed analysis. The results are presented as the mean ± SD from at least three independent experiments. The abbreviation ‘nd’ means that the determination was performed and the values were not detectable. The statistical significance of the differences between the values obtained for the WT and mutant strains was evaluated using one-way ANOVA and Dunnett’s post hoc test. The differences between the cells collected after 12 and 24 h of culture were evaluated using the *t*-test for independent samples. The values were considered significant at a *p-*value < 0.05. Used designations: * *p* < 0.05, ** *p* < 0.01, *** *p* < 0.001 comparing mutants vs. WT strain; ## *p* < 0.01, ### *p* < 0.001 comparing cells after 12 h vs. 24 h of culture.

Intracellular NADPH production in yeast cells during fermentative metabolism is based on enzymes such as glucose-6-phosphate dehydrogenase (Zwf1p), 6-phosphogluconate dehydrogenase (Gnd1p and Gnd2p), and aldehyde dehydrogenase 6 (Ald6p) [20]. The absence of any one of them forces the cells to activate an alternative pathway for NADPH production. However, although the goal is the same, both the alternative pathways and the cellular consequences of their activation may differ. For analysis of this issue, three isogenic mutants were selected: Δ*zwf1*, Δ*gnd1*, Δ*ald6*, and the wild-type strain. Glucose metabolism is not without significance in this case; therefore, the studies were performed after 12 h of culture, when the glucose concentration in the medium was approximately 1.8% (fermentative metabolism), and for comparison, after 24 h of culture, when the glucose was almost exhausted (after the metabolic shift from fermentation toward respiration; respiratory metabolism) [29,30]. The first analyses included the level of gene expression, protein content, and activity of these enzymes in the mutant strains compared to the wild-type strain. In the Δ*zwf1* mutant, the expression of the *GND1* and the *ALD6* genes was downregulated in comparison to the wild-type strain after 12 h of culture. This effect was also observed after 24 h of culture, but only for the *GND1* gene (Figure 1A). In turn, a lower level of the *ZWF1* gene expression and a higher level of the *ALD6* gene expression was demonstrated in the Δ*gnd1* mutant in comparison to the wild-type strain after 12 h of culture. There were no differences in these gene expressions in comparison to the wild-type strain after 24 h of culture (Figure 1A). In the Δ*ald6* mutant, a lower level of the *ZWF1* gene expression and a higher level of the *GND1* gene expression in comparison to the wild-type strain after 12 h of culture were shown. The expression of the *ZWF1* and the *GND1* genes was upregulated in comparison to the wild-type strain after 24 h of culture (Figure 1A). Furthermore, the expression of both the *ZWF1* gene and the *ALD6* gene was upregulated and the expression of the *GND1* gene was downregulated in the wild-type strain after the metabolic shift from fermentation toward respiration in comparison to fermentative metabolism (Figure 1A). These results show that yeast cells can compensate for the lack of enzymes responsible for cytosolic NADPH generation by changing the expression of selected genes. This is particularly evident in the case of the Δ*gnd1* and Δ*ald6* mutants. The expression of the *ALD6* gene is upregulated in the Δ*gnd1* mutant, allowing higher production of NADPH via aldehyde dehydrogenase 6. In turn, the Δ*ald6* mutant has upregulated expression of the *ZWF1* and *GND1* genes, which enables it to produce a large amount of NADPH in the pentose phosphate pathway.

The level of gene expression indicated the direction of the changes; therefore, it was examined whether the consequences of the lack of enzymes responsible for fermentation-dependent NADPH generation are also visible at the level of changes in the protein content and their enzymatic activity. To determine the Zwf1p, Gnd1p/Gnd2p, and Ald6p content, the Western blot method with anti-glucose-6-phosphate dehydrogenase, anti-6-phosphogluconate dehydrogenase, and anti-yeast aldehyde dehydrogenase antibodies was employed. Analysis of individual enzyme activities showed that the activity of glucose-6-phosphate dehydrogenase (Zwf1p) was decreased in the Δ*gnd1* mutant and increased in the Δ*ald6* mutant after 12 h of culture, in both cases relative to the wild-type strain (Figure 1C). This effect was also observed after 24 h of culture, but only for the Δ*gnd1* mutant (Figure 1C). In turn, a higher level of Zwf1 protein was only shown in the case of the wild-type strain after the metabolic shift from fermentation toward respiration in comparison to fermentative metabolism (Figure 1B). A higher level of Zwf1 protein may result from the upregulated expression of the *ZWF1* gene in this strain (Figure 1A). In the *S. cerevisiae* yeast, there are two isoforms of 6-phosphogluconate dehydrogenase. Gnd1p is the major isoform of this enzyme, accounting for about 80% of the activity, and Gnd2p is the minor isoform [27]. In our previous studies, we showed that the activity of 6-phosphogluconate dehydrogenase is reduced to the level of about 73% and 12% in the Δ*zwf1* and Δ*gnd1* mutant, respectively [13].

Our results confirm these observations (Figure 1D). The decreased activity of 6-phosphogluconate dehydrogenase in the Δ*zwf1* mutant may result from the lower Gnd1p/Gnd2p content (Figure 1B) and downregulation of the *GND1* gene expression (Figure 1A). On the other hand, the activity of Gnd1p/Gnd2p was increased in the Δ*ald6* mutant after both 12 and 24 h of culture (Figure 1D). The increased activity of this enzyme may be connected to the much higher Gnd1p/Gnd2p content (Figure 1B) and significant upregulation of the *GND1* gene expression in this mutant (Figure 1A). Furthermore, it was shown that after 24 h of culture, the activity of aldehyde dehydrogenase was very high in all the tested strains (Figure 1E). This may result from upregulation of the *ALD6* gene expression, but only in the case of the wild-type strain and the Δ*zwf1* and the Δ*gnd1* mutants (Figure 1A). It is worth noting, that despite the lack of *ALD6* gene expression (Figure 1A) and the Ald6p content (Figure 1B), increased activity of aldehyde dehydrogenase in the Δ*ald6* mutant was shown (Figure 1E). This indicates that the Δ*ald6* mutant produces/activates other aldehyde dehydrogenases similar to the Ald6p structure and properties (Figure S1), and with NAD(P)^+^-dependent aldehyde dehydrogenase activity (Figure 1E), which enables the cells to survive. The presented results demonstrate that the yeast cells can compensate for the lack of enzymes responsible for the cytosolic production of NADPH by changing the content of selected proteins and/or their enzymatic activity.

### 2.2. Changes in the Level of NADP(H) and NADPH/NADP^+^ Ratio in the Absence of Enzymes Responsible for Fermentation-Dependent NADPH Generation

**Figure 2:**
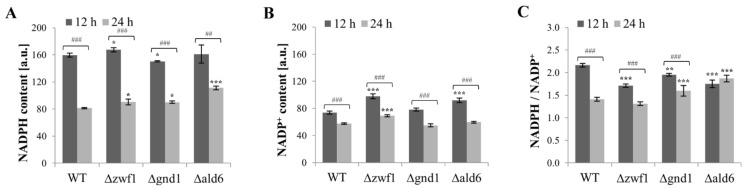
The content of the individual pyridine nucleotide cofactors NADPH (**A**) and NADP^+^ (**B**), and the NADPH/NADP^+^ ratio (**C**), was assessed in the cells of the wild-type (WT) strain and mutants Δ*zwf1*, Δ*gnd1*, Δ*ald6*. The analyses were performed after 12 h (fermentative metabolism) and 24 h of culture (condition after metabolic shift from fermentation toward respiration). The results are presented as the mean ± SD from at least three independent experiments in each case. The statistical significance of the differences between the values obtained for the WT and mutant strains was evaluated using one-way ANOVA and Dunnett’s post hoc test. The differences between the cells collected after 12 and 24 h of culture were evaluated using the *t*-test for independent samples. The values were considered significant at a *p*-value < 0.05. Used designations: * *p* < 0.05, ** *p* < 0.01, *** *p* < 0.001 comparing mutant vs. WT strain; ## *p* < 0.01, ### *p* < 0.001 comparing cells after 12 h vs. 24 h of culture.

The NADP(H) content depends on the glucose concentration in the culture medium and the type of metabolism (fermentative or respiratory) [16,31]. The level of NADPH and NADP^+^ in the Δ*zwf1* mutant was higher in comparison to the wild-type strain. This effect was observed after both 12 and 24 h of culture (Figure 2A,B). In the Δ*gnd1* mutant, the NADPH content was lower in comparison to the wild-type strain and the NADP^+^ content was at the same level as in the wild-type strain after both 12 and 24 h of culture (Figure 2A,B). In the Δ*ald6* mutant, the NADPH content after 12 h of culture was at the same level as in the wild-type strain and the NADP^+^ content was higher in comparison to the wild-type strain. In turn, after 24 h of culture, only the NADPH content was higher in this mutant in comparison to the wild-type strain (Figure 2A,B).

The level of NADPH and NADP^+^ was reduced after the metabolic shift from fermentation toward respiration (12 h vs. 24 h) in all the tested strains (Figure 2A,B). Both the content of pyrimidine cofactors and the relationship between them are important for maintaining redox homeostasis. The NADPH/NADP^+^ ratio was decreased in all the tested mutants compared to the wild-type strain after 12 h of culture (Figure 2C). On the other hand, the NADPH/NADP^+^ ratio was increased in the Δ*gnd1* and the Δ*ald6* mutants compared to the wild-type strain after 24 h of culture (Figure 2C). These results demonstrate that the lack of Zwf1p, Gnd1p, and Ald6p enzymes causes a disturbance in intracellular redox homeostasis, and these changes are strictly dependent on the type of metabolism.

### 2.3. Changes in the Level of GSH and GSSG, and GSH/GSSG Ratio in the Absence of Enzymes Responsible for Fermentation-Dependent NADPH Generation

Considering the role of NADPH in cellular redox homeostasis, and also the complexity of the system responsible for its maintenance, the analysis and interpretation of the results related to the changes in the NADP(H) level or the NADPH/NADP^+^ ratio also require reference to the changes in the GSH and GSSG level as the main intracellular redox buffer.

**Figure 3:**
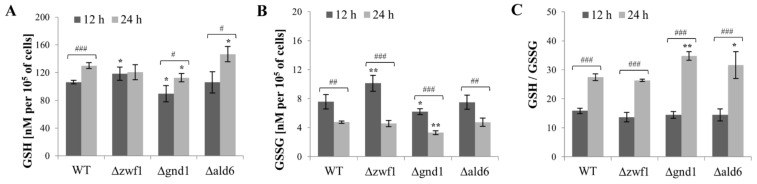
The content of the individual glutathione forms GSH (**A**) and GSSG (**B)**, and the GSH/GSSG ratio (**C**), were assessed in the cells of the wild-type (WT) strain and mutants Δ*zwf1*, Δ*gnd1*, Δ*ald6*. The analyses were performed after 12 h (fermentative metabolism) and 24 h of culture (condition after metabolic shift from fermentation toward respiration). The results are presented as the mean ± SD from at least three independent experiments in each case. The statistical significance of the differences between the values obtained for the WT and mutant strains was evaluated using one-way ANOVA and Dunnett’s post hoc test. The differences between the cells collected after 12 and 24 h of culture were evaluated using the *t*-test for independent samples. The values were considered significant at a *p-*value < 0.05. Used designations: * *p* < 0.05, ** *p* < 0.01 comparing mutant vs. WT strain; # *p* < 0.05, ## *p* < 0.01, ### *p* < 0.001 comparing cells after 12 h vs. 24 h of culture.

**Figure 4:**
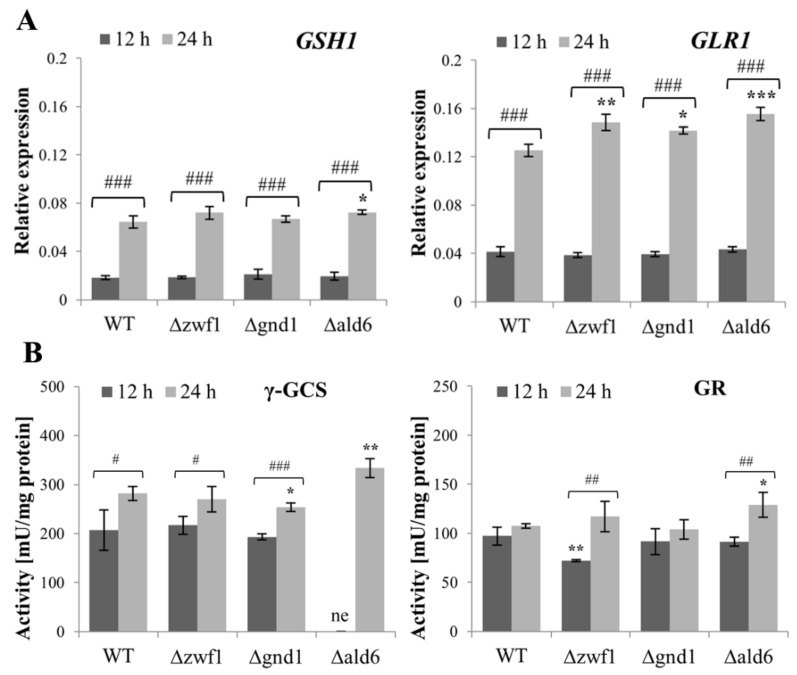
*GSH1* and *GLR1* gene expression and γ-GCS and GR enzyme activity in the wild-type (WT) and Δ*zwf1*, Δ*gnd1*, and Δ*ald6* mutant strains after 12 and 24 h of culture. *GSH1* and *GLR1* (**A**) gene expressions were determined by qPCR assay with TaqMan probes. The relative gene expression was calculated with the -ΔC_T_ method for comparison of the expression of one gene in all the tested strains. The activity of the γ-GCS and GR enzymes (**B**) in the whole-cell protein extracts was determined. The activity of γ-GCS was measured by the reaction coupled with lactic dehydrogenase and pyruvate kinase (PK/LDH enzymes mixture for determination of ADP) by the decrease of NADH absorbance at λ = 340 nm using a Varian Cary 50 spectrophotometer. The GR activity was determined by the rate of NADPH absorbance decrease at 340 nm using a Varian Cary 50 spectrophotometer. The results are presented as the mean ± SD from at least three independent experiments. The abbreviation ‘ne’ means that the sample was not estimated. The statistical significance of the differences between the values obtained for the WT and mutant strains was evaluated using one-way ANOVA and Dunnett’s post hoc test. The differences between the cells collected after 12 and 24 h of culture were evaluated using the *t*-test for independent samples. The values were considered significant at a *p*-value < 0.05. Used designations: * p < 0.05, ** p < 0.01, *** p < 0.001 comparing mutant vs. WT strain; *# p <* 0.05, *## p <* 0.01, *### p <* 0.001 comparing cells after 12 h vs. 24 h of culture.

The level of GSH and GSSG was higher in the Δ*zwf1* mutant in comparison to the wild-type strain, although this effect was only observed after 12 h of culture (Figure 3A,B). A higher level of GSH is not a result of changes in the *GSH1* gene expression or activity of γ-glutamylcysteine ligase (γ-GCS), which catalyzes the first step of GSH synthesis (Figure 4A,B). In turn, the higher level of GSSG in this mutant may result from the decreased activity of glutathione reductase (GR), which catalyzes the reduction of GSSG to GSH (Figure 4A,B). The GSH/GSSG ratio for the wild-type strain and the Δ*zwf1* mutant was at a similar level (Figure 3C). In the Δ*gnd1* mutant, the level of GSH and GSSG was lower in comparison to the wild-type strain after both 12 and 24 h of culture (Figure 3A,B). A lower level of GSH may result from the decreased activity of γ-GCS, but this effect was observed mainly after 24 h of culture (Figure 4B). The GSH/GSSG ratio for the Δ*gnd1* mutant after 12 h of culture was at a similar level as for the wild-type strain and the Δ*zwf1* mutant. In turn, after 24 h of culture, the GSH/GSSG ratio for this mutant was higher in comparison to the wild-type strain (Figure 3C). In the Δ*ald6* mutant, the level of GSH was higher in comparison to the wild-type strain, but only after 24 h of culture. The higher level of GSH may result from the upregulated expression of both the *GSH1* gene and the *GLR1* gene and the increased activity of γ-GCS and GR (Figure 4A,B). As a result of these changes, the GSH/GSSG ratio in the Δ*ald6* mutant after 24 h of culture was higher in comparison to the wild-type strain (Figure 3C). It is worth noting that the expression of the *GSH1* and *GLR1* genes was upregulated and the activity of γ-GCS and GR was increased after the metabolic shift from fermentation toward respiration (12 h vs. 24 h) in all the tested strains (Figure 4A,B). The consequences of these changes were a higher level of GSH, a lower level of GSSG, and an increased GSH/GSSG ratio (Figure 3A–C). The presented results demonstrate that changes in the level of GSH and/or GSSG may be adaptive responses to the absence of enzymes responsible for NADPH production. Moreover, these results show that the type of metabolism may also influence the contents of GSH and GSSG, and the GSH/GSSG ratio.

### 2.4. The Growth of Yeast Cells in the Absence of Zwf1p, Gnd1p, and Ald6p

**Figure 5:**
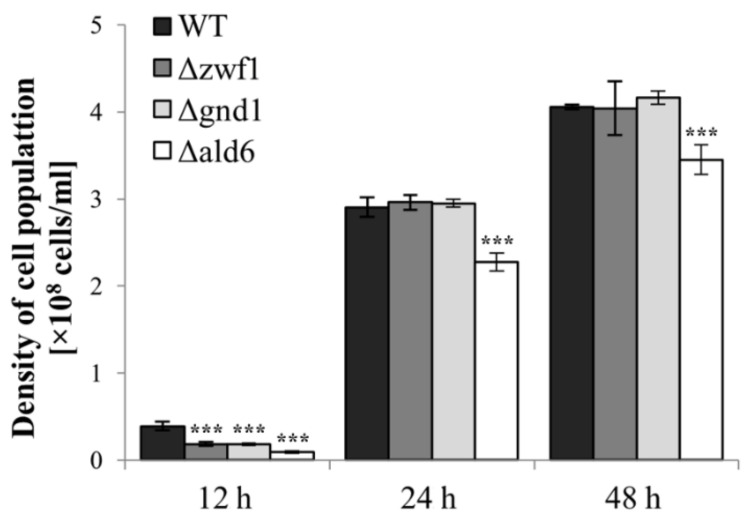
The growth of the wild-type (WT) and Δ*zwf1*, Δ*gnd1*, and Δ*ald6* mutant strains after 12, 24, and 48 h of culture in the liquid YPD medium. The growth expressed as the cell density (number of cells per mL) was estimated with a microscopic Malassez cell counter. The results are presented as the mean ± SD from at least three independent experiments. The statistical significance of the differences between the values obtained for the WT and mutant strains within a given time of culture, i.e., 12, 24, and 48 h of culture, was evaluated using one-way ANOVA and Dunnett’s post hoc test. The values were considered significant at a *p-*value < 0.05. Used designations: *** *p* < 0.001 comparing mutant vs. WT strain.

The ability of cells to grow is determined by environmental conditions, but above all, by the physiological efficiency of the cells. The absence of the analyzed enzymes, but also a cellular strategy to compensate for them, may influence cellular efficiency and thus the ability to grow. We have previously shown that disruption of the *ZWF1* and the *GND1* genes caused a decrease in the reproductive capacity of the yeast mutants only in the exponential phase of culture [13]. In this study, we confirmed these results for the Δ*zwf1* and Δ*gnd1* mutants but also added the result regarding the growth of the yeast strain lacking aldehyde dehydrogenase 6. It was shown that after 12 h of culture, the density of the cell population (number of cells per mL) of the Δ*zwf1*, Δ*gnd1*, and Δ*ald6* mutants was approximately 54%, 58%, and 80% lower, respectively, in comparison to the wild-type strain (Figure 5). In turn, after 24 and 48 h of culture, no differences in the density of the cell population of the wild-type strain, the Δ*zwf1* mutant, and the Δ*gnd1* mutant were observed (Figure 5). In the case of the Δ*ald6* mutant, the achieved cell population density was lower by approximately 20% and 18% after 24 and 48 h of culture, respectively (Figure 5). These results demonstrate that the lack of Zwf1p, Gnd1p, and Ald6p affects the growth of yeast cells, especially during the exponential phase of culture.

### 2.5. Changes in Intracellular Acidification and Extracellular pH in the Absence of Enzymes Responsible for Fermentation-Dependent NADPH Generation

In the yeast *S. cerevisiae*, organic acids, including acetic acid, are typical co-products of alcoholic fermentation. They can be used as a carbon source in respiratory metabolism; thus, in undisturbed conditions, acetic acid is not treated as a toxic compound, although it is constantly exported to the extracellular environment. The accumulation of metabolic-derived acetic acid occurs in parallel with glucose consumption and becomes maximal in the stationary phase when ethanol re-consumption is accompanied by acetate formation [32]. However, a disruption in cell metabolism may lead to changes in the acetic acid concentrations that trigger adaptive responses in cells. The final effect for the cell is a combined function of acetic acid production, intracellular accumulation, and extracellular pH, affecting both the export and uptake of acetic acid [33].

**Figure 6:**
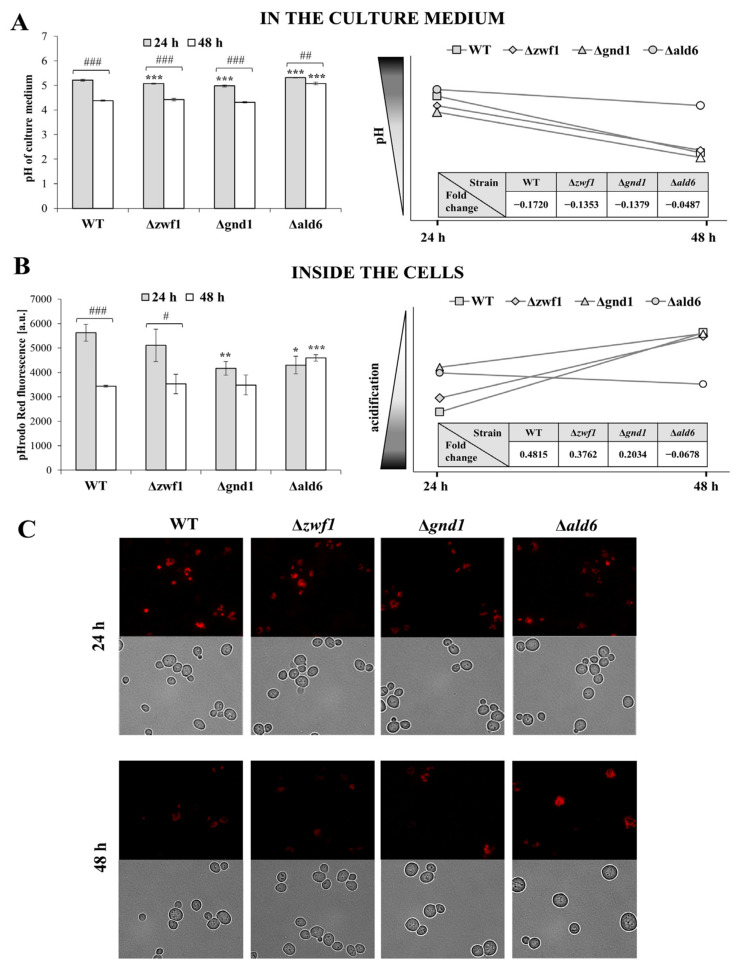
The pH of the culture medium and intracellular pH of the wild-type (WT) and Δ*zwf1*, Δ*gnd1*, and Δ*ald6* mutant strains after 24 and 48 h of culture in the liquid YPD medium. The pH of the culture medium (**A**) was measured in the supernatant (medium after the appropriate time of culture) using a laboratory pH meter. The intracellular pH (**B**) was determined with pHrodo Red fluorescent dye by fluorescence measurements using a Tecan Infinite 200 microplate reader at λex = 550 nm and λem = 590 nm. The results are presented as the pH or the values of fluorescence are presented as arbitrary units as well as the pH fold changes. The results of the pH values or values of fluorescence are presented as the mean ± SD from at least three independent experiments. The statistical significance of the differences between the values obtained for the WT and mutant strains was evaluated using one-way ANOVA and Dunnett’s post hoc test. The differences between the cells collected after 12 and 24 h of culture were evaluated using the *t*-test for independent samples. The values were considered significant at a *p-*value < 0.05. Used designations: * *p* < 0.05, ** *p* < 0.01, *** *p* < 0.001 comparing mutant vs. WT strain; # *p* < 0.05, ## *p* < 0.01, ### *p* < 0.001 comparing cells after 12 h vs. 24 h of culture. The vacuole visualization (**C**) using a fluorescence microscope Olympus BX-51 equipped with the DP-72 digital camera and cellSens Dimension v1.0 software. The microscopic images present typical results from the duplicate experiment. Magnification 1000×.

The analysis of intracellular acidification (Figure 6B,C) and extracellular pH (Figure 6A) shows that deletion of a particular gene connected with fermentation-dependent NADPH generation differentially influences pH homeostasis. The highest changes were noticed in the case of the Δ*ald6* and the Δ*gnd1* mutants, while the deletion of the *ZWF1* gene did not show any major disturbances compared to the changes observed in the wild-type strain (Figure 6). Considering that effective changes in the analyzed parameters occur after a longer culture, the presented results include data after 24 and 48 h of culture. After 24 h of culture, the intracellular acidification of both the Δ*ald6* and the Δ*gnd1* mutants was significantly lower in comparison to the wild-type strain (Figure 6B). The extracellular pH in the case of these strains was also changed, but in the opposite direction. The pH of the medium was higher, compared to the wild-type strain, in the case of the Δ*ald6* mutant and lower in the case of the Δ*gnd1* mutant (Figure 6A). After 48 h of culture, differences in pH homeostasis between the Δ*gnd1* and the wild-type strain were not further observed (Figure 6). In general, after 48 h of culture, intracellular acidification decreased (Figure 6B,C), which was accompanied by a decrease in extracellular pH (Figure 6A). This may result from that acetic acid generated during fermentation being successively exported outside the cell. This is not observed in the case of the Δ*ald6* mutant, in which the intracellular acidification does not decrease and the extracellular pH changes only slightly. The difference in intracellular acidification is also observed in the microscopic images; for example, in the case of the Δ*ald6* strain, many tiny vacuoles can be noted, the acidity of which does not change with the culture time (Figure 6C). To better illustrate the level of interdependent changes in intra- and extracellular acidity, the fold changes in these parameters for the analyzed strains during the culture are presented. The difference in the acidity changes of the Δ*ald6* mutant compared to the wild-type strain was confirmed again (Figure 6). The differences noted for the Δ*ald6* mutant with a high probability result from lower acetate production resulting in lower intracellular acidification but simultaneously in no need to remove the produced acetate from the cell, which results in the higher pH of the medium. However, it should be underlined that such an effect is not physiological, and this may also contribute to the weaker growth of the Δ*ald6* mutant cells (Figure 5).

### 2.6. Hypersensitivity to Acetic Acid in the Absence of Zwf1p and Gnd1p

**Figure 7:**
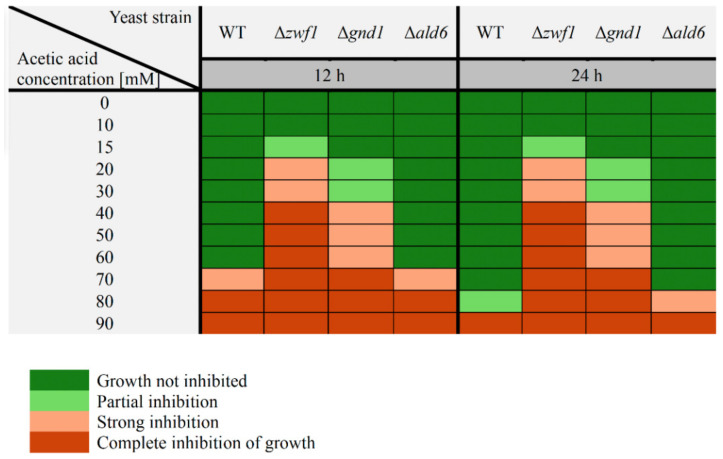
The growth of the wild-type (WT) and Δ*zwf1*, Δ*gnd1*, and Δ*ald6* mutant strains for 12 and 24 h of culture in the solid YPD medium supplemented with acetic acid in the concentration range from 0 to 90 mM. The growth was estimated by the spotting test. The colony growth was recorded after 48 h. Successive spots initially contained 50,000, 5000, 500, and 50 cells. The sensitivity of cells to acetic acid is presented by a suitable color scale: dark green—growth not inhibited; light green—growth partially inhibited; light red—strong inhibition of growth; dark red—growth completely inhibited.

Acetic acid belongs to the short-chain hydrophilic weak acids. The addition of acetic acid to the growth medium causes changes in the surrounding and intracellular environment. Stress induced by weak acids leads to internal acidification of yeast cells. Therefore, the ability to grow or maintain viability at a high concentration of weak acid may reflect the ability of cells to maintain their internal pH [34]. This study showed that both the Δ*zwf1* mutant and the Δ*gnd1* mutant were hypersensitive to acetic acid in comparison to the wild-type strain (Figure 7). Partial inhibition at 15 and 20–30 mM and strong inhibition of growth at 20–30 and 40–60 mM concentrations of acetic acid were shown in the Δ*zwf1* and the Δ*gnd1* mutants, respectively. In turn, complete growth inhibition was demonstrated at 40 and 70 mM concentrations of acetic acid in these mutants (Figure 7). No increased sensitivity to acetic acid was shown in the Δ*ald6* mutant in comparison to the wild-type strain (Figure 7). Strong and complete inhibition of growth was demonstrated, respectively, at 70 and 80 mM concentrations of acetic acid in both the wild-type strain and the Δ*ald6* mutant (Figure 7). A slightly higher sensitivity to acetic acid of the cells with fermentative metabolism (after 12 h of culture) in comparison to the cells after metabolic shift from fermentation toward respiration (after 24 h of culture) was shown, but only in the case of the wild-type strain and the Δ*ald6* mutant (Figure 7).

## 3. Discussion

### 3.1. Strategies for Maintaining Proper NADPH Level in the Case of PPP-Dependent NADPH Generation Disorders

Continuous adaptation to changing external conditions, counteracting threats related to the presence of reactive oxygen species or xenobiotics, and maintaining redox homeostasis are necessary for the proper functioning of cells and organisms. The glutathione system plays a key role in these processes, but the significant role of pyrimidine cofactors, especially NADPH, cannot be ignored. The main source of NADPH in *S. cerevisiae* yeast during growth in a medium with glucose is the pentose phosphate pathway. Glucose-6-phosphate dehydrogenase (Zwf1p) and 6-phosphogluconate dehydrogenase (Gnd1p and Gnd2p) are responsible for the reduction of NADP^+^ to NADPH in this pathway. The lack of any of these enzymes has several consequences for the cells and requires the activation of alternative pathways that enable NADPH production. In our previous study, we have shown that the Δ*zwf1* mutant shows changes in the cellular redox status caused by disorders in NADPH generation [13]. Reducing the ratios NADPH/NADP^+^, NADPH/NAD^+^, and (NADPH/NADP^+^)/(NADH/NAD^+^) compared to the wild-type strain causes significant disturbances of several anabolic reactions in this mutant. This applies in particular to biosynthetic processes, leading to a reduction in the growth ability of the cell population in the exponential phase of culture as well as a reduction in the reproductive potential of individual cells of the Δ*zwf1* mutant. These changes were not significant in the case of the stationary phase of culture and for the total lifespan of the cells ([13] and Figure 5 in this study). The growth retardation of cells with the deleted *ZWF1* gene has been observed previously in the case of both baker’s and wine yeast [35,36]. NADPH is essential for cells; therefore, the absence of the first enzyme of the PP pathway, responsible for its synthesis, does not mean the absence of this in the Δ*zwf1* mutant. Our studies have shown that the Δ*zwf1* mutant has even higher levels of NADPH and NADP^+^ in comparison to the wild-type strain after both 12 and 24 h of culture (Figure 2). Moreover, in the absence of the Zwf1p, the *GND1* gene expression was downregulated, and the Gnd1p/Gnd2p content and activity were decreased, in each case in comparison to the wild-type strain (Figure 1A–C). These results are consistent with previous reports that Zwf1p is the rate-limiting enzyme of the PP pathway and its absence causes the downregulation of other enzymes in this pathway ([13,37]; Figure 1A). One possible pathway for an alternative production of NADPH involves the aldehyde dehydrogenase 6 (Ald6p). It has been shown that deletion of the *ZWF1* gene results in increased acetate and oxyglutarate production [38]. However, in our studies, the expression of the *ALD6* gene was downregulated (Figure 1A), and the aldehyde dehydrogenase activity increased only slightly (Figure 1C), indicating that such compensation did not play a key role in this mutant. What is more, an increase in the *ALD6* gene expression and/or Ald6p activity would result in a higher level of acetic acid, and consequently, changes in the intracellular acidification and extracellular pH, but it was only observed to a small extent in the case of the Δ*zwf1* mutant (Figure 6). Furthermore, it was shown that the Δ*zwf1* mutant is hypersensitive to acetic acid (Figure 7) and another weak organic acid [39]. A strong negative correlation between fluxes through the PP pathway and acetate synthesis was also identified [40]. These arguments suggest that the Δ*zwf1* mutant cells activate a different and less obvious pathway of NADPH generation. As we postulated recently, NADPH may also be formed by hydrogen transfer between two pyridine coenzyme systems of NADH/NAD^+^ and NADP^+^/NADPH or reversible exchanges of NADH and NADP^+^ to NAD^+^ and NADPH [13]. Yet another option is to increase mitochondrial production by shifting cellular metabolism toward being more respiratory. The possibility of mitochondrial production in the Δ*zwf1* mutant is very likely due to the following premises: (i) stressful conditions and disorders demanding higher ATP formation to cope with the stress [39]; (ii) yeast cells can produce NADPH in a mitochondrial manner due to the activity of Pos5p (mitochondrial NADH kinase) or mitochondrial NADP^+^-dependent dehydrogenases (Idp1p; Mae1p; Ald4p or Ald5p) [41]; (iii) mitochondrial activity mainly in the TCA cycle produces a high level of reduced NADH, which can be used by Pos5p to generate NADPH [42]; and (iv) NADPH generated during growth on glucose is mostly required for biosynthesis reaction, whereas NADPH generated in nonfermentable carbon sources is necessary for both antioxidants systems and biosynthesis [43].

Analyses of other PP pathway enzymes have shown that although they are in the same pathway, the strategy for the production of NADPH in the alternative pathway is different and depends on the gene that was deleted. In the yeast strain with the disruption of the 6-phosphogluconate dehydrogenase 1 gene (Gnd1p, a major isoform of this enzyme, responsible for approximately 80% of its activity), it was shown that the *ZWF1* gene expression was downregulated and the Zwf1p activity was decreased in the Δ*gnd1* mutant in comparison to the wild-type strain (Figure 1A,C). This is the next proof that the activity of Zwf1p may be inhibited by the product of its reaction. Despite this, the activity of Zwf1p and Gnd2p (although at a significantly lower level) still allows for conversion in the PP pathway and generation of a low level of NADPH. Moreover, in the Δ*gnd1* mutant, the upregulation of the *ALD6* gene expression was demonstrated (Figure 1A). This indicates that this mutant may compensate for the lack of Gnd1p by increased production and/or activity of Ald6p. The consequence of this is a higher production of acetic acid in the cells and rapid release outside the cell, which was shown as a decreased pH of the culture medium (Figure 6). However, this solution cannot be used on a large scale because the Δ*gnd1* mutant is hypersensitive to acetic acid (Figure 7). This may be one of the reasons for the lower NADPH content and decreased NADPH/NADP^+^ ratio in this mutant (Figure 2). Previous results showed a reduction in the NAD^+^/NADH, NADP^+^/NADH, and (NADPH/NADP^+^)/(NADH/NAD^+^) ratios but not the NADPH/NADP^+^ and NADPH/NAD^+^ ratios in the Δ*gnd1* mutant in comparison to the wild-type strain [13], which may be caused by differences in the metabolic state of the cells. The results show that the lack of Gnd1p causes a disturbance in the intracellular redox homeostasis, which may cause a reduction in the growth ability of the cell population in the exponential phase of culture but not in the case of the stationary phase of culture as well as the total lifespan of the cells ([13] and Figure 5 in this study).

### 3.2. Strategies for Maintaining Proper NADPH Level in the Absence of the ALD6 Gene

The conversion of acetaldehyde to acetate by aldehyde dehydrogenase 6 is also an important means of NADPH generation. When the *ALD6* gene is deleted, acetate is formed by the mitochondrial dehydrogenases Ald4p and Ald5p. In connection with the fact that the *ALD4* gene is strongly repressed by glucose, it seemed that only the *ALD5* gene, strongly expressed in the exponential phase of growth during fermentation, could perform this function. However, Saint-Prix et al. have shown that Ald4p can compensate for the lack of Ald6p in yeast grown on glucose, and this compensation requires the induction of *ALD4* gene transcription [44]. In the Δ*ald6* mutant, the *ALD4* gene is derepressed even in the presence of glucose, both the *ALD4* transcripts and Ald4p protein are detected during the fermentative metabolism, and the activity of K^+^-activated aldehyde dehydrogenase is increased [44,45]. However, compensating for the lack of the *ALD6* gene has several consequences for the cells, including a reduction of acetate formation by 70–77% during growth on the YPD medium with 5% glucose [44]. Less acetic acid also means lower production of NADPH. To compensate for this deficiency, the Δ*ald6* mutant increases NADPH production in the PP pathway. It was shown that the expression of the *ZWF1* and the *GND1* genes was upregulated and the activity of Zwf1p and Gnd1p/Gnd2p was increased in the Δ*ald6* mutant in comparison to the wild-type strain (Figure 1A–C). As a result, the Δ*ald6* mutant has a similar or even higher level of NADPH compared to the wild-type strain after 12 and 24 h of culture, respectively (Figure 2A). It was also demonstrated that a higher level of NADP^+^ causes a decrease in the NADPH/NADP^+^ ratio (Figure 2B,C). Consistent with our results are data [32] presenting increased xylitol production in the Δ*ald6* strain, which the authors explain by the higher Zwf1p activity and the compensatory effect of the NADPH supply.

### 3.3. Consequences of Strategies Compensating for NADPH Deficiency in the Δald6 Mutant

The decrease in acetate production is associated with a change in extracellular and intracellular acidification (Figure 6). It has been shown that impairment in acetate generation in the Δ*ald6* mutant results in lower intracellular acidification after 24 h, but at the same time, with lower acetate export from the cells to the medium, which is confirmed by only a slight change in the pH of culture medium between 24 and 48 h (Figure 6A). The pH of the culture medium decreases by approximately 0.85 between 24 and 48 h in the wild-type strain and by only approximately 0.25 in the case of Δ*ald6* mutant (Figure 6A). The strong relationship between the intracellular and extracellular pH was also confirmed by the results of the analysis of these parameters during the culture. The intracellular acidification and pH of the medium significantly changed between 24 and 48 h of culture in the wild-type strain (fold changes were appropriately −0.1720 and 0.4815); at the same time, they hardly changed in the Δ*ald6* mutant (fold changes were appropriately −0.0487 and −0.0678) (Figure 6). Lower acetate production and unusual changes in intracellular acidification in the case of the Δ*ald6* mutant may also have long-term effects. It is suggested that yeast resistance to weak organic acids and acetic acid tolerance may depend largely on the maintenance of cytosolic pH [46]. Unexpectedly, it was observed that besides the decreased rate of growth (Figure 5), cells of the Δ*ald6* mutant show no increased acetic acid sensitivity (Figure 7). The reason for this may be that the low level of acetic acid produced in the Δ*ald6* strain cells allows them to survive relatively high exogenous concentrations of acetic acid. However, the increased sensitivity of cells of this strain is still observed in the case of other organic acids, including propionic acid [34]. This lack of increased sensitivity to acetic acid of the Δ*ald6* mutant (Figure 7) can also be the result of changes in the transport of acetic acid inside the cells, which is strictly dependent on the environmental conditions, including the extracellular pH. When the extracellular pH is below 4.76 (acetic acid pKa), acetic acid exists mainly in the undissociated form, which can freely diffuse through the plasma membrane, but when the extracellular pH is higher, acetic acid is present mainly as acetate anions, entering the cells through the two main proton symporters, Jen1 and Ady2 [33,47].

The issue of the pH balance seems to be important for the Δ*ald6* mutant cells. Also, for this reason, the intracellular pH is an element of a complex signaling system that combines the availability of nutrients and the rate of cell growth [48]. In conditions of full glucose availability, its metabolism through fermentation leads to the production of ethanol but also organic acids, which, when removed from the extracellular environment, cause its rapid acidification. Acidification of the extracellular environment can be a source of stress, but an alkaline pH is also unfavorable for the cells because similar values of extracellular pH and cytosolic pH can result in disruption of the absorption of nutrients and ions. For this reason, it is crucial to strictly control the pH of the cytosol but also of organelles such as the vacuoles, which are a site of degradation but also a place of metabolite storage, including amino acids and ions necessary for the physiological efficiency of the cell [49]. Both the degradation of macromolecules and the storage of metabolites in the vacuole require maintaining its acidic environment. Recent studies show that cells dynamically regulate the pH of the vacuole in individual phases of the cell cycle. It has been shown that the inability to dynamically regulate vacuolar pH may influence the release of amino acids from the vacuole, e.g., arginine [50]. This may result in a reduction in the translational capacity, which in turn may directly affect the growth rate. Such an observation might help explain why the Δ*ald6* mutant strain shows a decreased growth rate (Figure 5) despite the restoration of the NADPH level to the wild-type strain’s level (Figure 2A). This is also indicated by the fact that in the case of these cells, there were no significant changes in the extracellular and cytosolic pH (Figure 6), which may lead to a disturbance in the dynamic regulation of the vacuolar pH.

### 3.4. Mutual Interaction between GSH/GSSG and NADPH/NADP^+^ Couples in Maintaining Redox Homeostasis

The cellular redox homeostasis requires a constant balance between the reduced and oxidized forms of the GSH/GSSG and NADP(H) redox couples. Currently, more and more papers demonstrate the connection and balancing of the GSH/GSSG and NADPH/NADP^+^ systems. The possibility of rapid equilibration of the glutathione and NADP(H) redox couples is indicated by work carried out both on yeast cells [6,14] and cell lines [51]. Additionally, the use of specific redox sensors indicates that the cytosolic NADP(H) and glutathione redox potential can be similar (the range of −290 to −340 mV for E_NADP(H)_ and the range of −300 to −320 mV for E_GSH_) [17]. Undeniably, the relationship between the redox couples is largely due to the role that NADPH plays in maintaining the reduced state of glutathione. Due to its high concentrations (millimolar levels), glutathione is considered a primary redox couple and the main source of cellular-reducing equivalents. In the case of glutathione, especially important for the cells is to limit its oxidation resulting in GSSG generation. The strict control of the GSSG level results from the fact that relatively small changes in the GSSG concentration would lead to significant changes in the redox potential [52,53]. This is even more important in the case of mitochondrial matrix glutathione oxidation. This is the outcome that (i) cytosolic-synthesized glutathione must be imported into the mitochondria; (ii) the inner mitochondria membrane is largely impermeable to GSSG and the matrix glutathione pool is isolated; (iii) the glutathione-caring system is more developed in the cytoplasm; and (iv) matrix glutathione oxidation promotes cell death [14,52,53,54]. The importance of preventing the accumulation of GSSG is demonstrated by the fact that cells possess several cooperative systems to maintain a properly reduced glutathione pool. These include GSSG reduction systems (i.e., glutathione reductase–Glr1p; thioredoxin and glutaredoxin systems–the most important role of Trx2p and Grx2p), pathways for reducing glutathione oxidation (e.g., hyperoxidation of Prx1p) or compartmentalization of GSSG (e.g., Ycf1p-mediated sequestration of GSSG excess to the vacuole) [1,14,52,53,54,55]. Our results significantly support previously postulated conclusions, and at the same time, help to better understand the relationship between the glutathione and NADP(H) redox couples. First, the balance between the GSH/GSSG and NADPH/NADP^+^ systems was confirmed (Figure 2 and Figure 3). This is mostly visible when the culture conditions are changed (metabolic shift from fermentation toward respiration) and the increase in the GSH/GSSG ratio (Figure 3) is accompanied by a decrease in the NADPH/NADP^+^ ratio (Figure 2). Activation of aerobic respiration requires the provision of significant amounts of reducing equivalents. Since glutathione plays a major reducing role in the cell, the greatest changes are observed in its level (Figure 3 and Figure 4). The activation of respiratory metabolism results in a significant increase in the synthesis (increased *GSH1* expression and γ-GCS activity) and reduction of glutathione (increased *GLR1* expression and GR activity) (Figure 4). This results in a significant increase in the GSH/GSSG ratio (Figure 3C). To prevent disruption of redox homeostasis, the second redox couple, the NADPH/NADP^+^ ratio, is reduced (Figure 2C). This reduction in the case of switching to respiratory metabolism leads to a decrease in the NADPH level (Figure 2A), and additionally, to an increase in the expression of glutathione reductase, which is an important consumer of NADPH (Figure 4A). A high glutathione level and high GSH/GSSG ratio under respiration-inducing conditions have already been observed [56]. The paper of Tello-Padilla et al. [56] showed a significantly higher GSH level and GSH/GSSG ratio under caloric restriction than their levels observed in cells cultured in a medium with different glucose concentrations. In turn, an increase in the expression and activity of glutathione reductase (Figure 4) observed during the metabolic shift from fermentation toward respiration can be connected to the fact that Glr1p is limiting for GSSG reduction in the mitochondrial matrix [54].

Analyzing the changes in the redox systems observed under full fermentation conditions (12 h of culture), it can be postulated that maintaining an appropriate GSH/GSSG ratio is crucial for cellular redox homeostasis, which is achieved in all the analyzed strains (Figure 3C). This may be obtained by changes in the level of NADPH (Figure 2), which, on the one hand, may be the result of different NADPH biosynthesis capabilities in the analyzed strains, but on the other hand, may also be the result of different cell demands for NADPH. In the case of the Δ*zwf1* mutant after 12 h of culture, a significantly increased level of GSSG (Figure 3B), partly resulting from the decreased glutathione reductase activity (Figure 4B), can be observed. This level of GSSG may be a signal for the cell to increase NADPH production (Figure 2A), which can be used by thioredoxins and alternative GSSG-reducing systems or used as a specific counterbalance. The increased level of NADP^+^ (Figure 2B) may indicate that the emerging NADPH pool is being consumed. The increased level of GSSG in the Δ*zwf1* mutant after 12 h of culture is also counteracted by increasing the level of GSH (Figure 3A), although this is not a direct result of the increased expression and activity of γ-glutamylcysteine ligase (Figure 4), but this may be achieved through greater uptake from the culture medium. The fact that the actions taken by the cell in the absence of the *ZWF1* gene are effective is demonstrated by (i) maintaining an appropriate GSH/GSSG ratio (Figure 3C), (ii) the expression and activity of glutathione reductase increases after 24 h of culture (Figure 4), and (iii) the GSSG level after 24 h of culture drops to the level observed for the wild-type strain (Figure 3B). A different situation occurs in the case of the Δ*gnd1* mutant. In this strain, a significantly reduced level of GSSG compared to the wild strain was observed after both 12 and 24 h of culture (Figure 3B). This results in no need to have a high level of GSH; hence, the level of GSH is reduced (Figure 3A), the activity of γ-glutamylcysteine ligase is lowered (Figure 4B), but the GSH/GSSG ratio is maintained (Figure 3C). These observations are also related to the changes observed in the NADP(H) pool. A low level of GSSG and maintained GSH/GSSG potential will not force the generation of a high level of NADPH or its high consumption (Figure 2). In the meantime, in the case of the Δ*ald6* strain, especially after 24 h of culture, no equilibration between the NADP(H) and GSH/GSSG redox couples is observed. There is a significant increase in the level of glutathione synthesis and reduction (Figure 4), which significantly increases the level of reduced glutathione as well as the GSH/GSSG ratio (Figure 3). Importantly, this is accompanied by a significant increase in NADPH synthesis (Figure 2A), mainly through the PP pathway (Figure 1), and an increase in the NADPH/NADP^+^ ratio (Figure 2C). Although such dysregulation does not increase oxidative stress, it has negative consequences that are manifested by reduced cell growth of the Δ*ald6* strain (Figure 5). The lack of compensation between the GSH/GSSG and NADPH/NADP^+^ redox couples will probably lead to a state of reductive stress, which may reduce cell growth, e.g., by disrupting the proper folding of proteins in the ER, leading to activation of the UPR (unfolded protein response) and ER stress [51,57].

## 4. Materials and Methods

### 4.1. Yeast Strains and Growth Conditions

In the study, the wild-type strain (WT) BY4742 *MAT*α *his3 leu2 lys2 ura3*, which was the control strain, and three mutant strains isogenic to BY4742, Δ*zwf1* YNL241c::kanMX4, Δ*gnd1* YHR183w::kanMX4, and Δ*ald6* YPL061w::kanMX4, were used (EUROSCARF, Scientific Research and Development GmbH, Oberursel, Germany). The yeast was grown in the standard liquid YPD medium (1% Yeast Extract, 1% Yeast Bacto-Peptone, 2% glucose) on a rotary shaker at 150 rpm and at a temperature of 28 °C. Cells from an overnight preculture were used for the experiments. A density of 1 × 10^5^ cells/mL in a total volume of 20 mL of medium was used as a starting point. Individual analyses were performed after 12, 24, or 48 h of culture. The cell density (number of cells per mL) was estimated with a microscopic Malassez cell counter.

### 4.2. RNA Samples

The RNA samples were obtained using the GeneMATRIX Universal RNA Purification Kit (EURx, Gdansk, Poland) according to the manufacturer’s protocol. After 12 and 24 h of culture, cells were centrifuged, washed twice with sterile water, and suspended to a density of 5 × 10^7^ cells/mL in the spheroplast buffer (1 M sorbitol, 0.1 M EDTA, 0.1% β-mercaptoethanol) containing lyticase (250 U per sample) for 30 min at 30 °C. The resultant spheroplasts were used for RNA isolation. The RNA samples were stored at −80 °C and each of them was thawed only once. Four independent biological replicates were prepared for each strain. The concentration and purity of the RNA samples were measured using a Tecan Infinite 200 microplate reader equipped with a NanoQuant Plate using a 260 nm/280 nm ratio.

### 4.3. Real-Time PCR

A total of 500 ng of RNA previously treated with DNase I (Roche, Mannheim, Germany) for 60 min at 25 °C (10 U per 1 µg RNA) was used for the reverse transcription. To synthesize cDNA, SuperScript IV VILO Master Mix (Invitrogen, Thermo Fisher Scientific, Waltham, MA, USA) was applied according to the manufacturer’s protocol, and the samples were stored at −20 °C until use. Real-time PCR was performed using Roche LightCycler 96 equipment and TaqMan chemistry. Briefly, the cDNA sample was diluted and mixed with TaqMan Fast Advanced Master Mix and TaqMan Gene Expression Assays (Applied Biosystems, Life Technologies, Pleasanton, CA, USA). The *ZWF1*, *GND1*, *ALD6*, *GSH1*, and *GLR1* gene expression levels were tested. The *ACT1* gene was used as an internal control. The relative gene expression was calculated with the -ΔC_T_ method for comparison of the expression of different genes in the same strain or one gene in all the tested strains.

### 4.4. Protein Extraction

The cells collected after 12 and 24 h of culture were centrifuged, washed twice with sterile water, and suspended in cold homogenization buffer (20 mM phosphate buffer, pH 6.8, containing 1 mM EDTA, 0.2% DTT, and 1 mM PMSF). Then, the biomass was disrupted with 0.5 mm glass beads in 6 cycles of 30 s, with intervals for cooling the sample on ice, and then centrifuged (14,000× *g*, 15 min, 4 °C). The supernatants were transferred to new tubes and immediately frozen at −80 °C. Four independent biological replicates were prepared for each strain. The protein concentration was determined using the Bradford method.

### 4.5. Western Blot

The protein samples were separated by SDS-PAGE and then transferred to nitrocellulose membrane (PVDF Western Blotting Membranes, Roche) by semidry immunoblotting (BioRad, Hercules, CA, USA). After blocking with PBST buffer (PBS, 0.1% Tween 20) containing 3% nonfat milk, the following primary antibodies were used: anti-glucose-6-phosphate dehydrogenase (1:2000, ab87230, Abcam, Cambridge, UK), anti-6-phosphogluconate dehydrogenase (1:2000, ab125863, Abcam), anti-yeast aldehyde dehydrogenase (1:4000, ab182893, Abcam), and anti-yeast alcohol dehydrogenase (1:4000, ab34680, Abcam). The respective proteins were detected after incubation with the horseradish peroxidase-conjugated secondary antibodies (1:10,000, 111,035,003, Jackson ImmunoResearch, West Grove, PA, USA) with a SuperSignal West PICO Chemiluminescent Substrate (Pierce Biotechnology, Waltham, MA, USA) according to the manufacturer’s protocol. Yeast alcohol dehydrogenase (Adh1p) was used as an internal control. The images were captured using an Azure c300 Imaging System.

### 4.6. Enzyme Assays

The total activity of the PP pathway dehydrogenases (sum of both the glucose-6-phosphate dehydrogenase (Zwf1p) and 6-phosphogluconate dehydrogenase (Gnd1p and Gnd2p) activities), and separately, the 6-phosphogluconate dehydrogenase activity, was determined spectrophotometrically by measuring the rate of NADP^+^ reduction at 340 nm according to the method of Tian et al. [58], with the authors’ own modifications. In turn, the Zwf1p activity was calculated by subtracting the activity of Gnd1p and Gnd2p from the total enzyme activity. To obtain the total dehydrogenase activity, 0.2 mM NADP^+^, 0.4 mM D-glucose-6-phosphate, and 0.4 mM 6-phosphogluconate as reaction substrates were used. The substrates were added to 100 mM Tris-HCl buffer, pH 8.0, containing 1 mM MgCl_2_. The addition of 5 µL cell extract (2 mg of protein per mL) initiated the reaction. In turn, to obtain the Gnd1p and Gnd2p activity, only 0.2 mM NADP^+^ and 0.4 mM 6-phosphogluconate were used as reaction substrates. The kinetics of the absorbance increase was recorded using a Tecan Infinite 200 microplate reader at λ = 340 nm. The activity was expressed in arbitrary units.

The aldehyde dehydrogenase (ALD) activity was determined with an Aldehyde Dehydrogenase Activity Colorimetric Assay Kit (Sigma-Aldrich, Poznan, Poland) according to the manufacturer’s protocol, with its own modification. In this assay, acetaldehyde was oxidized by ALD generating NADH, which reacts with a probe, producing a colorimetric product proportional to the ALD activity presented in the whole-cell protein extracts (1 mg of protein per mL). The absorbance was measured against the blank for 36 min every 2 min using a Tecan Infinite 200 microplate reader at λ = 450 nm. The activity was expressed in arbitrary units.

The glutathione reductase (GR) activity was determined by the rate of NADPH absorbance decrease at 340 nm using a Varian Cary 50 spectrophotometer. The reaction mixture contained 50 mM phosphate buffer pH 7.0, 0.5 mM DTPA, and 80 µM NADPH. To exclude unspecific NADPH oxidation, a 1 min incubation of the reaction mixture with the protein extract sample was applied before the addition of 2 mM GSSG (final concentration) and then the absorbance was recorded. The GR activity was calculated with an extinction coefficient of 6.22 mM^−1^ cm^−1^ and expressed as U per mg protein.

The γ-glutamate-cysteine ligase (γ-GCS) activity was determined according to the method of Watanabe et al. [59], with its own modification. The rate of ATP usage during the reaction of γ-GCS was measured by the reaction coupled with lactic dehydrogenase and pyruvate kinase (PK/LDH enzymes mixture for determination of ADP; P0294 Sigma-Aldrich, Poznan, Poland) by the decrease of NADH absorbance at λ = 340 nm using a Varian Cary 50 spectrophotometer. The coupled reaction was run at the temperature of 37 °C in the mixture containing 50 mM HEPES buffer, pH 7.0, 12 mM glutamate, 4 mM MgCl_2_, 30 mM ATP, 12.5 mM cysteine, 0.5 mM phosphoenolpyruvate, 0.9 U PK, 1.6 U LDH, 0.1 mM NADH and protein extract sample. Appropriate control reactions (without sample and PK/LDH) were performed and subtracted. The γ-GCS activity was calculated with an extinction coefficient of 6.22 mM^−1^ cm^−1^ and expressed as U per mg protein.

### 4.7. Determination of NADP(H) Content

The NADP(H) content in the yeast cells was assessed with the NADP/NADPH-Glo Assay kit (Promega, Madison, WI, USA) according to the manufacturer’s protocol, with its own modifications [13]. After 12 and 24 h of culture, the cell density (number of cells per mL) was determined using the Malassez chamber. The cells were centrifuged and washed twice with sterile water and suspended in a PBS buffer. A density of 2 × 10^6^ cells/mL was used for the assay. From each culture, the cell suspension was transferred to Eppendorf tubes and incubated in 1:1 ratio with lysis solution (0.2 M NaOH with 1% DTAB (dodecyltrimethylammonium bromide) for 15 min with intense shaking. After incubation, the samples were split into separate tubes for measuring the NADP^+^ and NADPH. The next steps of the procedure were performed according to the manufacturer’s protocol. The luminescence signal proportional to the amount of NADP^+^ or NADPH was recorded for 3 h at 25 °C using a Tecan Infinite 200 microplate reader. The value of the blank was subtracted each time. The results were presented as the individual pyridine cofactors’ content and NADPH/NADP^+^ ratio.

### 4.8. Determination of Glutathione Content

The total glutathione (sum of both GSH and GSSG) and separate GSSG levels were determined in the yeast cells with the GSH/GSSG-Glo Assay (Promega, Madison, WI, USA) according to the manufacturer’s protocol, with its own modifications [13]. After 12 and 24 h of culture, the cell density (number of cells per mL) was determined using the Malassez chamber. The cells were centrifuged and washed twice with sterile water and suspended in a PBS buffer. A cell number of 5 × 10^5^ was used for the assay. From each culture, the cell suspension was added to a white flat bottom 96-well plate in duplicate, one for measuring the total glutathione and the second for measuring the GSSG level. The luminescence was recorded after 15 min using a Tecan Infinite 200 microplate reader. The total glutathione and GSSG concentrations were read based on the standard curves, whereas the level of GSH was calculated by subtracting the GSSG from the total glutathione concentration (due to 1 mole of GSSG being generated by 2 moles of GSH, the values of GSSG were multiplied by 2).

### 4.9. Determination of pH of Culture Medium

The cells collected after 12, 24, and 48 h of culture were centrifuged and used for future analyses. The pH was measured in the supernatant (medium after the appropriate time of culture) using a Hach sensION+ PH3 laboratory pH meter. The pH measurement in each sample was performed in triplicate. The results were presented as the pH of the culture medium and as the pH fold changes.

### 4.10. Determination of Intracellular pH

The yeast cell pH was stained with pHrodo Red AM Intracellular pH Indicator (Thermo Fisher Scientific, Waltham, MA, USA) according to the manufacturer’s protocol, with suitable modifications. After 24 and 48 h of culture, the cells were centrifuged, washed twice with PBS buffer, and resuspended in Live Cell Imaging Solution (LCIS: 20 mM HEPES buffer, pH 7.4, 140 mM NaCl, 2.5 mM KCl, 1.8 mM CaCl_2_, 1 mM MgCl_2_). pHrodo Red AM Intracellular pH Indicator was added to a final concentration of 2.5 mM (500 mM stock solution). After 30 min of incubation, the cells were washed with LCIS and resuspended in fresh LCIS. The intracellular pH was determined by fluorescence measurements using a Tecan Infinite 200 microplate reader at λex = 550 nm and λem = 590 nm and visualized by fluorescence microscopy at appropriate wavelengths. The microscopic images were captured at 1000× magnification with the Olympus BX-51 microscope equipped with the DP-72 digital camera and cellSens Dimension v1.0 software. The values of the fluorescence measurements were presented as arbitrary units but also as acidification fold changes according to manufacturer’s information, demonstrating that higher fluorescence values mean lower intracellular pH.

### 4.11. Spotting Test

The cells collected after 12 and 24 h of culture were centrifuged, washed with sterile water, and diluted to provide a suspension of 10^7^, 10^6^, 10^5^, and 10^4^ cells/mL. Aliquots of 5 µL for each suspension were inoculated onto a solid YPD medium containing 10, 15, 20, 30, 40, 50, 60, 70, 80, and 90 mM concentrations of acetic acid. Freshly prepared stock solution of acetic acid (10 M stock in sterile water) was added to sterile media after cooling to approximately 55 °C. The colony growth was inspected after 48 h.

### 4.12. Statistical Analysis

The results are presented as the mean ± SD from at least three independent experiments. The statistical analysis was performed using the Statistica 13.3 software. The statistical significance of the differences between the wild-type strain and the mutants was evaluated using one-way ANOVA and the Dunnett’s post hoc test. The differences between the cells collected after the analyzed time of culture were compared using a *t*-test. The values were considered significant at a *p < 0.05*.

## 5. Conclusions

The lack of enzymes responsible for fermentation-dependent NADPH generation causes the disorder of the cellular redox homeostasis. To compensate for this deficiency and provide an adequate pool of NADPH, which is necessary for antioxidant systems and biosynthesis, cells use various strategies. However, the choice of compensation strategy has several consequences for cell functioning; for example, reduction of the reproductive capacity of cells and thus population growth, changes in intracellular acidification, and sensitivity to acetic acids. Redox homeostasis requires a constant balance between the reduced and oxidized forms of the GSH/GSSG and NADPH/NADP^+^ redox couples. Therefore, maintaining the proper pool of NADPH is crucial for the efficient reduction of glutathione and thus maintaining the optimal redox balance in cells. These findings are crucial for understanding cellular physiology as well as cells’ possibilities for adjusting to various circumstances. Additionally, it may be very beneficial in synthetic biology applications.

## Data Availability

The data generated and analyzed during this study are available from the corresponding author upon reasonable request.

## Supplementary Materials

The following supporting information can be downloaded at: https://www.mdpi.com/article/10.3390/ijms25179296/s1.
