# Supplementary material for: Strategies to Maintain Redox Homeostasis in Yeast Cells with Impaired Fermentation-Dependent NADPH Generation
Source: Int J Mol Sci. 2024 Aug 27;25(17):9296. doi: 10.3390/ijms25179296 (PMC11395483; doi:10.3390/ijms25179296)
Supplement: Supplementary file 1 [file ijms-25-09296-s001.zip › ijms-3168954-supplementary.pdf]

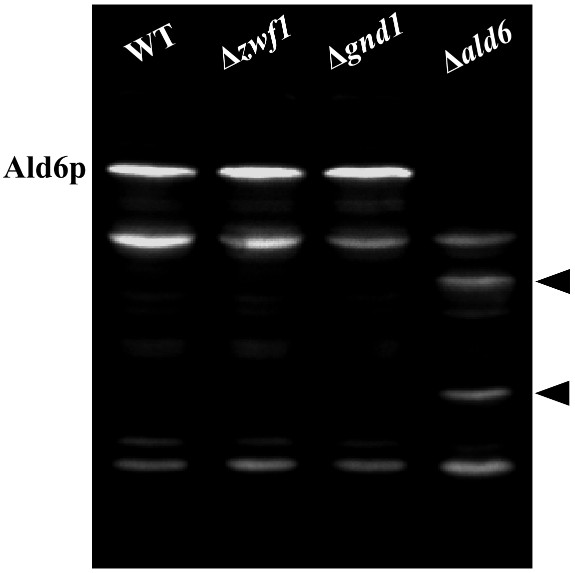

Figure S1. The aldehyde dehydrogenase 6 (Ald6p) in the wild-type strain (WT) and Δ*zwf1*, Δ*gnd1*, and Δ*ald6* mutants was detected by immunoblotting assay with the primary antibodies anti-yeast aldehyde dehydrogenase (1:4000) and with the horseradish peroxidase-conjugated secondary antibodies (1:10 000) with a chemiluminescent substrate. The arrows indicate additional proteins that appear in the Δ*ald6* mutant.

See also Figure 1
